# Quantitative set analysis for gene expression: a method to quantify gene set differential expression including gene-gene correlations

**DOI:** 10.1093/nar/gkt660

**Published:** 2013-08-05

**Authors:** Gur Yaari, Christopher R. Bolen, Juilee Thakar, Steven H. Kleinstein

**Affiliations:** ^1^Department of Pathology, Yale University School of Medicine, New Haven, CT 06511, USA, ^2^Bioengineering program, Faculty of engineering, Bar Ilan University, 5290002, Ramat Gan, Israel and ^3^Interdepartmental Program in Computational Biology and Bioinformatics, Yale University, New Haven, CT 06511, USA

## Abstract

Enrichment analysis of gene sets is a popular approach that provides a functional interpretation of genome-wide expression data. Existing tests are affected by inter-gene correlations, resulting in a high Type I error. The most widely used test, Gene Set Enrichment Analysis, relies on computationally intensive permutations of sample labels to generate a null distribution that preserves gene–gene correlations. A more recent approach, CAMERA, attempts to correct for these correlations by estimating a variance inflation factor directly from the data. Although these methods generate *P*-values for detecting gene set activity, they are unable to produce confidence intervals or allow for post hoc comparisons. We have developed a new computational framework for Quantitative Set Analysis of Gene Expression (QuSAGE). QuSAGE accounts for inter-gene correlations, improves the estimation of the variance inflation factor and, rather than evaluating the deviation from a null hypothesis with a *P*-value, it quantifies gene-set activity with a complete probability density function. From this probability density function, *P-*values and confidence intervals can be extracted and post hoc analysis can be carried out while maintaining statistical traceability. Compared with Gene Set Enrichment Analysis and CAMERA, QuSAGE exhibits better sensitivity and specificity on real data profiling the response to interferon therapy (in chronic Hepatitis C virus patients) and Influenza A virus infection. QuSAGE is available as an R package, which includes the core functions for the method as well as functions to plot and visualize the results.

## INTRODUCTION

Microarrays and RNA-seq have made simultaneous expression profiling of many thousands of genes across several experimental/clinical conditions widely accessible. However, interpreting the profiles from such large numbers of genes remains a key challenge. An important conceptual advance in this area was the shift from a focus on differential expression of single genes to testing sets of biologically related genes (1). The development of statistical tests to identify gene sets that are differentially expressed between groups (e.g. control and treatment) remains an active area of ongoing research [see e.g. (2–4)]. Gene sets are defined *a priori* as sharing some biologically relevant property (e.g. members of the same pathway, having a common biological function, presence of a binding motif, etc.). In addition to the obvious advantage in interpretability, a key benefit of analyzing gene sets compared with individual genes is that small changes in gene expression are unlikely to be captured by conventional single-gene approaches, especially after correction for multiple testing. This was demonstrated by (1), where an oxidative phosphorylation gene set was identified as downregulated in diabetic patients, even though none of the individual genes were downregulated by >20%. Another factor in the popularity of gene set analysis is the availability of publicly accessible databases, such as MSigDB (5), that contain easy-to-use and high-quality gene sets. Gene set analysis methods are generally used to test one of two null hypotheses, either (i) the genes in a set are not on average differentially expressed or (ii) the genes in a set are at most as differentially expressed as genes not in the set. Methods that test null hypothesis (i) are called self-contained, whereas those that test null hypothesis (ii) are called competitive (6). The advantages and disadvantages of each approach have been extensively debated, and each has a distinct interpretation. Self-contained tests assess the relevance of individual biological processes, whereas competitive tests seek to distinguish the most important biological processes from others that are less important. It has been suggested that self-contained tests be used as an initial screening that may be followed up with a competitive test (6). In both cases, the failure of most methods to account for gene–gene correlations has been recognized as a major effect that can produce high Type I error (6–13). The end result of current gene set methods is a *P*-value, which tests for differential expression of the set of genes as a unit. This focus on *P*-values restricts the ability to carry out post hoc comparisons and increase the likelihood of methodological flaws (14). This is especially limiting, as most existing methods are designed to compare only two groups (e.g. treatment and control). Although comparing a treatment response between two cohorts (e.g. the response to treatment with different drugs) can be done, each comparison requires its own setup, which is time-consuming and error-prone. *P*-values also do not lend themselves to intuitive visualizations, which can be an important part of interpreting gene set analysis results. The popularity of Gene Set Enrichment Analysis (GSEA) is due in part to the availability of an intuitive plot of gene set differential expression. The ‘Enrichment Plot’ [see Figure 2 in (15)] displays the positions of genes in the set in a ranked list of all the genes being measured. The ability to directly see that the individual genes in the set ‘tend’ to appear among the most differentially expressed genes (whether up- or downregulated) provides important corroboration for the *P*-value that is output. Gene sets that have significant *P*-values may be dismissed if the pattern does not appear biologically meaningful [see S3 in Figure 2 of (15)].

Since the development of GSEA (15), many approaches for testing the differential expression of gene sets have been proposed (2). The method proposed here, Quantitative Set Analysis of Gene Expression (QuSAGE), is unique in several aspects:

- Gene set differential expression is quantified by a full probability density function (PDF) rather than a single *P*-value.
- A variance inflation factor (VIF) is estimated from the data and used to modify this PDF to account for gene–gene correlation.
- Individual gene statistics are calculated using a Welch test formalism so that no assumption of equal variance of expression between control and treatment is required.

### Approach

**Figure 1.**
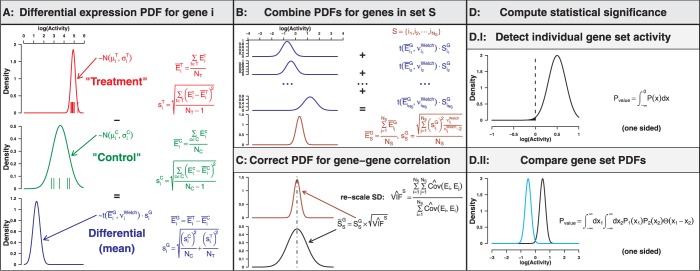
Overview of the steps to carry out QuSAGE.

Given a matrix of gene expression values, QuSAGE carries out four steps for each gene set of interest (see also Figure 1):

1. Compare individual gene expression values between two groups (paired or non-paired) to obtain a full PDF for differential expression of genes.
2. Combine the individual PDFs from within the gene set into a single ‘activity’ distribution using numerical convolution.
3. Correct the variance of the combined PDF to account for gene–gene correlation by calculating a VIF.
4. Compute statistical significance by comparing the PDF to a baseline value or to the PDF of another gene set.

## MATERIALS AND METHODS

### Estimate the PDF for differential expression of individual genes

A ‘t-test’ formalism is used to estimate the difference in the mean expression of each gene between two groups (e.g. ‘control’ and ‘treatment’, which we use throughout the article as generic labels for sample groups). For gene *i*, expressions are annotated as 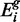 [where 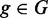 are the indexes of the samples that belong to a single group *N_G_*, and *G* is one of control (*C*) or treatment (*T*)]. Unbiased estimates for the mean and standard deviation within each group are then calculated, respectively, by:
(1)
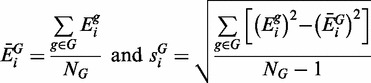

To model the difference in mean expression for gene *i* between the two groups, we define:
(2)
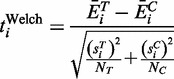

According to Welch, 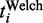 approximately follows Student’s *t*-distribution with ν degrees of freedom, where ν is given by:
(3)
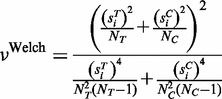

In cases where a pooled variance approach is taken, the standard deviation for the difference in mean expression is calculated from each of the group standard deviations as:
(4)
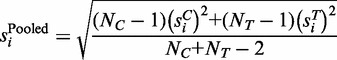

and 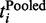 is defined as:
(5)
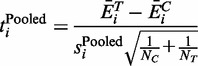

which follows the Student’s *t*-distribution with 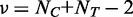 degrees of freedom.

To analyze paired samples, the difference in individual gene expression is first calculated for each pair of samples (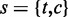):
(6)
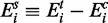

from which the mean and standard deviation for the difference in expression between the groups are given respectively by:
(7)
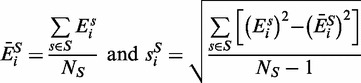

and we define:
(8)
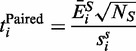

which follows Student’s *t*-distribution with 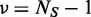 degrees of freedom.

The full PDF for the difference in expression between the groups is generated for each gene by scaling the Student’s *t*-distribution by the standard deviation. By scaling, we arrive at a distribution for the difference in expression, rather than the *t*-statistic itself. This distribution is shifted to be centered around 0 (by subtracting the expected difference in expression) and sampled at a fixed number of points (4096 by default). The range for sampling is determined by the degrees of freedom such that at most 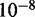 of the cumulative distribution at the tails are excluded (i.e. assumed to be 0). For example, when 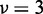, the range is 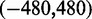, and when 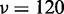, the range is 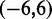.

### Combine individual PDFs using convolution

Given a gene set, a single PDF for the difference in expression is generated by using numerical convolution applied to the individual gene PDFs. This step builds on our previously published algorithm (16), by taking advantage of the fact that the distributions here are real and symmetric (Student’s *t*-distributions). Briefly, a Fast Fourier Transform is calculated for each individual gene PDF to arrive at a k-component vector. The product of each component across all of the genes is then taken to arrive at a new k-component vector for the gene set. The real part of the resulting product is then transformed back to a PDF using a reverse Fast Fourier Transform and assured to be normalized and centered around zero. The mean of the combined PDF is simply the mean fold change of the individual genes. Technically, the output of this step is the PDF of the sum of differences in expressions over all genes in the gene set under the assumption that the genes are independent. To estimate the mean differential expression PDF, this distribution is scaled by a factor of 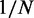, where *N* is the number of genes in the gene set.

### Account for gene–gene correlation

Correlation between genes in a set is taken into account by scaling the gene set PDF using a VIF. This is done, as by definition, the variance of the mean difference in expression for a set of *N* genes (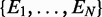) is:
(9)
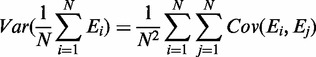



Up to this point, genes were assumed to be independent, which implies that 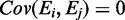 for 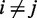. Therefore, the VIF is estimated as:
(10)
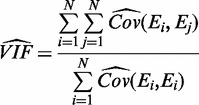

where 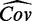 represents the unbiased covariance estimator, which is calculated for an individual group G as follows:
(11)
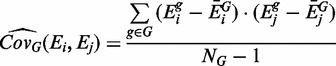

where 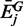 is the estimator for the mean [Equation (1)], 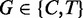 and *N_G_* is the size of the group (e.g. control or treatment). When using the Welch approximation, a VIF is estimated from the covariance of each individual group, and a single VIF is calculated as the mean of the VIFs for each group weighted by the group size.

When using a pooled variance approach, the covariance is given by:
(12)
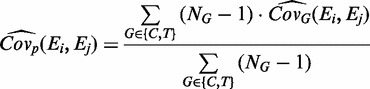

where 
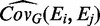
 is the covariance estimation for group *G* [Equation (11)]. Finally, having calculated a VIF for the gene set, the PDF for the difference in expression between the groups is scaled by a factor of (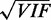). Thus, the standard deviation of the gene set PDF is increased when there is a mean positive correlation between genes in the set 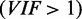.

### Using moderated statistics for individual gene differential expression

When estimating the difference in expression between groups for individual genes, many current studies use moderated statistics (e.g. ebayes in LIMMA and SAMtools). These new standard deviations can be integrated into QuSAGE as follows:

- The *t*-statistic [Equations (2), (8) and (5)] for each gene *i* is re-calculated using the new moderated standard deviation estimation (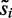). For methods that also moderate the degrees of freedom (ν), such as LIMMA, these new values should be used.
- The VIF calculation is adjusted by modifying the covariance matrix:
(13)
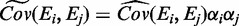

where 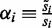 and 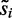 is the moderated standard deviation.

### Statistical significance of gene set activity

A *P*-value for detecting activity of a single gene set is calculated by comparing the gene set PDF to a baseline value using a one-sided test:
(14)
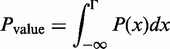

where Γ is the value to be compared against. For a ‘self-contained’ test, 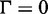, whereas a ‘competitive’ test is implemented by setting Γ to be the mean differential expression of genes not included in the gene set. A *P*-value for differences in expression between two gene set PDFs is calculated by:
(15)
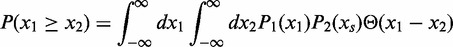

where 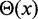 is the Heaviside step function, which equals 0 when 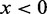, 1 when 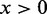 and 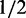 when *x* = 0.

### Data sets

#### Interferon therapy response

Gene expression data from three clinical studies of the response to interferon (IFN) therapy in chronic Hepatitis C virus patients were downloaded from the Gene Expression Omnibus (GEO): Study 1 (17) (GEO ID: GSE11190) included samples from both peripheral blood mononuclear cells (PBMCs) and liver, pre- and 4 h post-therapy, Study 2 (18) (GEO ID: GSE7123) included PBMC samples pre- and 1 day post-therapy, and Study 3 (19) (GEO ID: GSE11342) included PBMC samples pre- and 3 days post-therapy. In all studies, patients were defined as clinical responders if at least a 1000-fold decrease in the level of hepatitis C virus (HCV) RNA in the blood was observed 4 weeks post-IFN therapy. All other patients were considered to be clinical non-responders.

#### Influenza A virus infection response

Temporal gene expression data were downloaded for 17 healthy human subjects before and after they were challenged with live influenza A virus (H3N2/Wisconsin) (20) (GEO ID: GSE30550). Patients were defined as symptomatic or asymptomatic based on a standardized symptom scoring metric [as described in (20)], combined with nasopharyngeal viral titers after 24 h.

All data were normalized using the GCRMA package in R. For each data set, genes were removed if fewer than two samples had expression values greater than a threshold of 16 (indicating background expression). For the gene set analysis of these data, the set of IFN-stimulated genes (ISGs) was defined by (21).

## RESULTS

### Quantifying gene set activity

Given two groups to compare, gene set activity in QuSAGE is quantified by the mean difference in log expression of the individual genes that compose the set. A key feature of QuSAGE is the estimation of the full PDF for this activity. We start by computing the difference in expression for each individual gene under the common assumption that the log expression follows a normal distribution within each of the groups (control and treatment). This interval estimation problem is generally known as the Behrens–Fisher problem, and there are two main approaches to calculate the distribution (see ‘Materials and Methods’ section for details). In the first method (the pooled variance approach), the variances of the two groups are assumed to be equal, and the difference in the mean expression values is modeled as a Student’s t distribution. The second method, proposed by Welch (22), is also based on a Student’s t distribution but relaxes the assumption of equal variances. Most recent gene expression studies take the first approach and assume that the variances of the two groups are equal. For example, this assumption underlies the individual gene statistics in the widely used LIMMA package (23) in Bioconductor.

**Figure 2.**
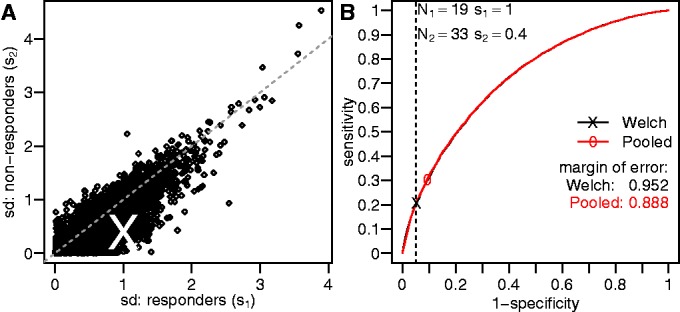
The impact of unequal gene expression variance across groups. (**A**) The standard deviation of individual gene expression values (points) was calculated using data from pre-therapy PBMC samples in a study of chronic HCV infection (18). Samples were divided into two groups depending on the clinical response to therapy, and separate standard deviations were calculated for each group. Equality is indicated by the dashed line. (**B**) ROC curves based on stochastic simulations (see text) for testing the difference between two groups using Welch’s approximation (black line) or the pooled variance approach (red line). The parameters for the stochastic simulations were based on the empirical data [indicated by a white x in (A)]. The X and 0 indicate the values for which 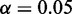.

The pooled variance approach can slightly improve sensitivity (if the equal variance assumption holds) and is easily compatible with linear models and Analysis of variance (ANOVA). However, this approach can be extremely biased when the assumption of equal variances is broken, which we find is often the case in many real gene expression data sets. To illustrate this fact, we turn to a series of clinical studies on the response of chronic HCV patients to IFN therapy, which we use as running examples throughout this article. Figure 2A plots the estimated standard deviation of 12 718 genes that were measured in one of these studies before the initiation of IFN therapy (18). In this case, the patients were classified by their clinical response to therapy, and it is clear that the standard deviation for most genes is higher in strong responders. To demonstrate the impact of these unequal variances, the sensitivity and specificity of both approaches were estimated using stochastic simulations based on the actual sample sizes (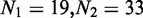) and standard deviations (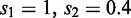) from an example gene (the X in Figure 2A). Specificity (1-false-positive rate) was calculated by sampling two groups from normal distributions with the same mean (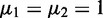), whereas the sensitivity (true-positive rate) was calculated by sampling similar distribution with different means (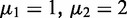). The results for each approach (Pooled variance and Welch) are summarized as receivers operating characteristic curves (Figure 2B). Although the two receivers operating characteristic curves lie on top of each other, the desired specificity (Type I error) for the pooled approach is biased leading to a significantly higher false-positive rate than the α level. Thus, we recommend the use of the Welch formalism for most cases, as there is little benefit, and significant potential disadvantages, to assuming that the variance of the ‘treatment’ measurements will be similar to the ‘control’.

### Accounting for gene–gene correlation

The expression measurements of individual genes in a set are almost always correlated, and the assumption of independence made by most existing gene set methods has been repeatedly criticized (6–13). Several approaches have been suggested to account for inter-gene correlations. A straightforward method is to estimate *P*-values by permuting the group labels of the samples. This is implemented by the widely used GSEA method (15). However, permutations are computationally intensive and perform poorly with small sample sizes (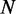 ≾ 7 per group), where the number of possible permutations is limited. A more recent approach, CAMERA, estimates a VIF directly from the data and uses this to correct for the error introduced by gene–gene dependencies in a set (13). QuSAGE adopts a similar approach but, unlike CAMERA, does not require the assumption of equal variances for all the genes in each set.

**Figure 3.**
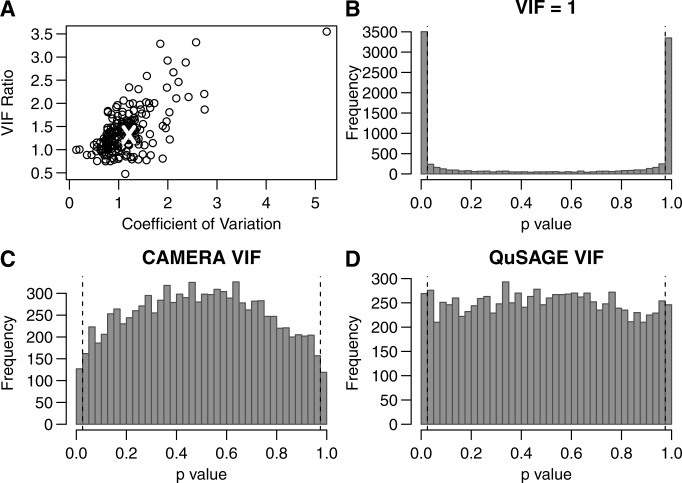
Comparison of methods to account for gene–gene correlations within a set. Gene expression data from a single homogenous group of samples [pre-therapy PBMC samples of poor responders from (18)] were randomly divided into two groups. (**A**) VIFs were calculated for 186 KEGG pathway gene sets (points) and the ISG gene set (white x) using CAMERA and QuSAGE. The ratio between these VIF estimates is plotted against the coefficient of variation of the standard deviations for individual genes in each set. (**B**) The random division of samples into two groups was repeated for 10 000 iterations and *P*-values were calculated for the activity of the ISG gene set. Gene–gene correlations were either (B) ignored (VIF = 1), (**C**) corrected using CAMERA or (**D**) corrected using QuSAGE. Type I errors for 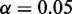 (indicated by the fraction of the distribution outside the vertical dashes lines) were (B) 0.685, (C) 0.02 and (D) 0.052.

**Figure 4.**
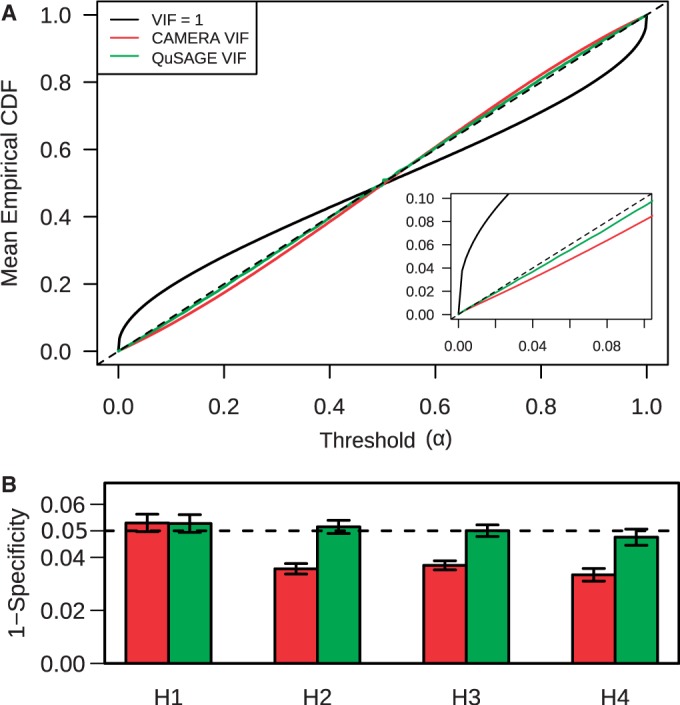
QuSAGE VIF estimation effectively controls the Type I error. (**A**) *P*-values were calculated for the activity of each pathway in the KEGG database (24) using the same data and approach as Figure 3B–D. Gene–gene correlations were either ignored (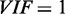) (black line), corrected for using CAMERA (red line) or corrected using QuSAGE (green line). The empirical cumulative distribution function (CDF) was calculated as the fraction of pathways with *P*-values below the indicated α threshold, with the dashed line indicating the specificity of an ideal test. The inset shows a closer look at the vicinity of 0. (**B**) The same procedure was repeated using four independent data sets containing healthy individuals [H1-4 that correspond to (25–28)]. The mean (±standard error) empirical CDF at 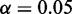 is plotted using VIF corrections from CAMERA (red) and QuSAGE (green).

The equal variances (across genes) assumption taken by CAMERA leads to a slightly more computationally efficient VIF calculation. However, this assumption is not valid for most gene sets, and its violation can greatly impact specificity. For example, we found that many Kyoto Encyclopedia of Genes and Genomes (KEGG) pathways contain genes with widely varying standard deviations. In these cases, the VIFs calculated by CAMERA and QuSAGE differ significantly (Figure 3A). To test whether QuSAGE improves specificity under these conditions, we generated data sets where genes are expected to have no true differential expression, but gene–gene correlations within the data would be preserved. In our first test, the set of pre-therapy samples from HCV patients who did not respond to therapy (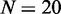 poor responders, a subset of non-responders) was divided randomly into two groups (‘treatment’ and ‘control’). This procedure was repeated 10 000 times and, in each permutation, a *P*-value was calculated for detecting ISG activity (21). We focused on the set of ISGs because these are biologically relevant for studying chronic HCV infection. As with many of the KEGG pathway gene sets, the genes in this set also have a wide range of standard deviations (across subjects, see Figure 3A). When calculating ISG activity, gene–gene correlations were either ignored (VIF = 1), corrected using CAMERA, or corrected using QuSAGE (with all other parts of the computation held fixed). As there is no difference between the permuted groups, our expectation is that the *P*-value distribution should be uniform. However, CAMERA often produced right-skewed *P*-value distributions (high type II error rate) compared with QuSAGE (see Figure 3C and D). Pathways where the VIF estimates were close resulted in similar *P*-value distributions for CAMERA and QuSAGE (data not shown). Both VIF estimation methods performed better than the independence assumption (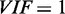), which has the highest Type I error (Figure 3B). To test whether these findings were generally true, we repeated this procedure using KEGG pathway gene sets. In Figure 4A, the mean empirical cumulative distribution function for the fraction of KEGG pathways with significant activity (of the 186 KEGG pathways) is plotted against the α level. When genes were assumed to be independent (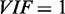), many more pathways were found to be significant than expected based on the α cutoff. In contrast, both CAMERA and QuSAGE tended to be conservative, with CAMERA generally over-correcting. Similar results were obtained using four independent data sets containing gene expression profiles from cohorts of healthy individuals (25–28) (Figure 4B). Thus, the VIF correction implemented in QuSAGE effectively controls the Type I error rate in the face of inter-gene correlations while providing improved accuracy and power compared with CAMERA. This estimation can be applied in both the pooled variance and Welch’s approaches to individual gene differential expression analysis.

### Visualizing gene set activity

Visualization of gene set activity can provide biological insights beyond the calculation of single *P*-values. The ability of QuSAGE to estimate full PDFs for gene set activity enables a number of useful figures to be generated. R functions to generate several of these are made available as part of the QuSAGE package.

#### Comparison of gene set activity across two cohorts

**Figure 5.**
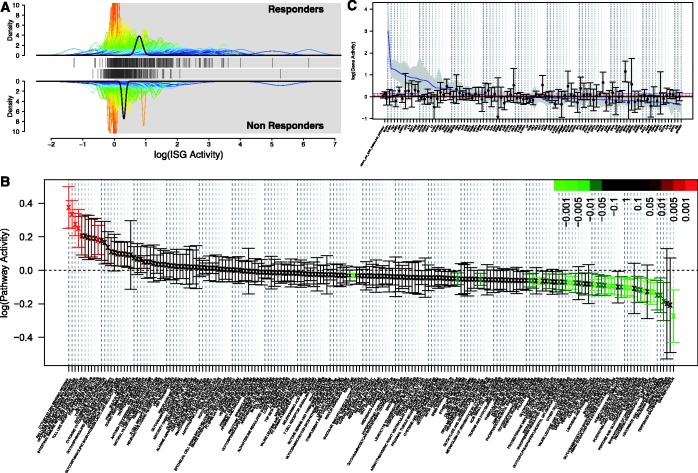
Visualization methods in QuSAGE. (**A**) The ISG response in HCV patients to IFN therapy is compared in both clinical responders (upper panel) and non-responders (lower panel). Differential expression PDFs (comparing post- and pre-therapy time-points) are shown for individual genes (thin curves color-coded by standard deviation), along with the aggregated estimate for the ISG pathway after taking into account gene–gene correlation (thick black curve). The mean differential expression for individual genes in the set are indicated as line barcodes between the two panels. (**B**) Summary of gene set activity (post- versus pre-therapy) among clinical responders for the 186 pathway gene sets in KEGG. For each pathway, the mean and 95% confidence interval are plotted and color-coded according to their False discovery rate (FDR)-corrected *P*-values when compared to zero. (**C**) Mean and 95% confidence interval for differential expression of individual genes in the KEGG JAK STAT SIGNALING pathway are shown for clinical responders (blue line and gray band) and non-responders (points and bars color-coded by FDR-corrected *P*-values for comparison with zero). Horizontal dashed lines indicate the mean differential expression for responders (blue) and non-responders (red). In all plots (A, B, C), data are taken from (17) (see MATERIALS AND METHODS section for details). R code to produce these plots is part of QuSAGE and includes options allowing many of the features to be customized.

Many experiments seek to compare the transcriptional response to perturbation across two (or more) cohorts. For example, in studying the response to IFN therapy in HCV patients, a key question is whether the blood transcriptional response (post- versus pre-therapy) differs depending on the patients’ overall response to therapy (responder versus non-responder based on viral load measurements). As the patients are treated with IFN, the set of ISGs is a natural set to consider. Using QuSAGE, activity of the set of ISGs defined by (21) was quantified in each cohort. These results are plotted in Figure 5a, which displays the activity PDF for the entire ISG set, along with the differential activity of the individual genes that compose the set. In this way, it can easily be determined whether the activity results from small changes in many genes, or large changes in just a few genes. In addition, the gene set activity for both cohorts are plotted together so that these can be directly compared. This comparison across patient cohorts can also be quantified by a *P*-value using functions supplied in the QuSAGE package (also see ‘Materials and Methods’ section), and such post-hoc analysis is another key advantage of QuSAGE.

#### Gene set screening summary

Although the ISG set is an obvious candidate to analyze when studying the response to IFN therapy, in many cases, we are interested in identifying changes in gene sets which might suggest new biology. This is most often accomplished by screening large numbers of pre-defined gene sets, such as the ones made available in MSigDB (5), at the same time. Figure 5B displays the treatment-induced activity of 186 pathway gene sets from the KEGG database. In this case, the mean and 95% confidence interval are extracted as summary measures of the full activity PDF. From this visualization, both induced and repressed pathways can be identified. The PDF for each pathway gene set can be compared with zero to obtain a *P*-value for detecting pathway activity (see ‘Materials and Methods’ section). These *P*-values, which can be corrected for multiple hypotheses testing using standard methods, are used to color-code each pathway based on significance. As expected from Figure 5A, the Jak–Stat pathway (which includes many ISGs) is significantly induced.

#### Detailed view of gene set activity

Another visualization, shown in Figure 5C, allows interrogation of the individual genes that compose a single gene set. This can be used to identify the subset of genes that drive activity. It may also be the case that the genes in a set are not homogeneous in the direction that they change as clearly seen in Figure 5C (see DISCUSSION section for further comments).

### QuSAGE improves sensitivity over existing methods

We compared the performance of QuSAGE with the existing GSEA and CAMERA approaches on two real data sets: profiling the response to IFN therapy (in chronic HCV patients) and Influenza A virus infection.

#### Application to IFN treatment response in HCV patients

QuSAGE was next applied to the analysis of the response to IFN therapy in patients infected with chronic HCV. Approximately 3 million people in the USA are chronically infected with HCV. IFN therapy is costly, poorly tolerated due to adverse side effects and ineffective in many patients. The ability to predict which patients are least likely to respond to IFN therapy would be a great help in clinical decision making and could allow alternate treatments to be explored.

Gene expression analysis in liver biopsies before and after the initiation of IFN therapy have suggested that clinical non-responsiveness is associated with a high baseline level of ISG expression, leading to a stunted response following IFN treatment (17,18,29). While promising, there are significant disadvantages to a liver biopsy compared with the use of a non-invasive blood test to predict the outcome of therapy. Unfortunately, the same study that identified significant differences in liver biopsies failed to find any response-related differences in the PBMC transcriptional profiles of the same patients (17). The lack of clinical response-related differences in PBMCs was also observed by (19), although an earlier study by this group suggested that overall gene induction following IFN therapy may be blunted in poor responders (18). These generally disappointing results call into question the potential of blood transcriptional profiles for predicting HCV treatment response. However, we recently found that chronic HCV infection does induce a blood (2) transcriptional signature that can be identified at the gene set level (30). This observation motivated us to reconsider previous studies of treatment response.

**Figure 6.**
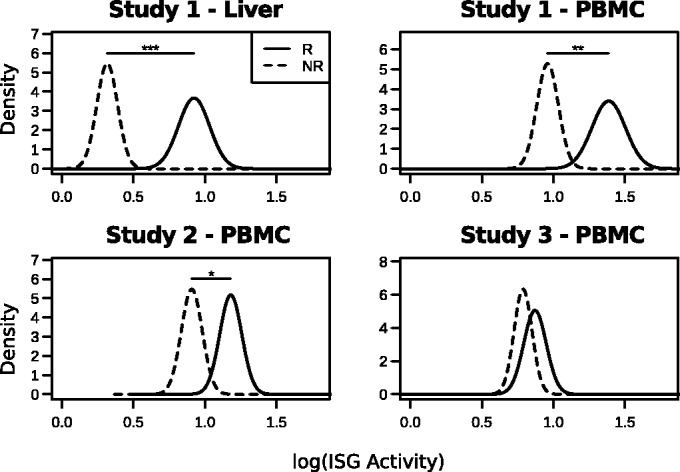
QuSAGE reveals significantly stronger activation of the IFN pathway in HCV therapy responders. ISG set differential expression was calculated for clinical responders (solid lines) and non-responders (dashed lines) by comparing gene expression measurements from matched post- and pre-treatment samples. Studies 1, 2 and 3 refer to (17–19), respectively. The activity PDFs for responders and non-responders were compared: Asterisk indicates 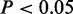, double asterisk indicates 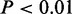 and triple asterisk indicates 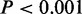.

QuSAGE was used to analyze genome-wide expression patterns in chronic HCV patients from three different clinical studies (17–19) (referred to here as Studies 1, 2 and 3, respectively). These studies each included microarray-based expression profiling of PBMCs pre- and post-IFN therapy. One of the studies also included liver biopsies from the same patients pre- and post-therapy (17). We first used QuSAGE to compare pre-therapy ISG expression levels between clinical responders and non-responders. Consistent with (17), pre-therapy ISG expression in the liver was significantly higher in non-responders (*P* < 0.001). As expected by previous studies, this association was not observed in the PBMC samples from any of the studies (data not shown), suggesting that pre-therapy ISG expression in PBMCs is unlikely to be a useful biomarker to predict therapy response. Next, we applied QuSAGE to estimate ISG set activity, defined as the difference between post- and pre-therapy samples. This activity was then compared between clinical responders and non-responders. Using this approach, we were able to reproduce the previous observation in Study 1 that clinical responsiveness was associated with significantly stronger up-regulation of ISG expression in the liver (Figure 6). Importantly, we also found evidence for this association in PBMCs in all three studies (Figure 6). While the difference was only statistically significant for Studies 1 and 2, this is likely explained by the later post-treatment sampling time of Study 3 as the magnitude of the difference decreased with the length of time between the pre- and post-treatment blood samples. These results are particularly important because a difference in IFN treatment response has not been previously observed in PBMCs, and it suggests that a blood test may be useful for predicting therapy response. However, this test is only useful if done within a short time after IFN treatment, as the signal disappears 3 days post-treatment. In addition, these results suggest that the blunted ISG response in non-responders is not solely due to pre-activation of the IFN pathway as previously suggested, since no differences in baseline ISG expression levels were observed for PBMCs. In addition to ISG activity, QuSAGE detects significant activity of many other pathways in patients responding to IFN therapy, even when the number of samples is small (e.g., 6 non-responders versus 10 responders in Figure 5B). In contrast, GSEA is unable to detect this activity since it depends on permutations to account for gene-gene correlations, which imposes a theoretical limitation in the possible *P* values that can be produced (Supplementary Figure S1). Overall, these results demonstrate the power of QuSAGE to discover new biology, and show that QuSAGE can be more sensitive than GSEA when sample sizes are small.

#### Application to influenza A virus infection response

**Figure 7.**
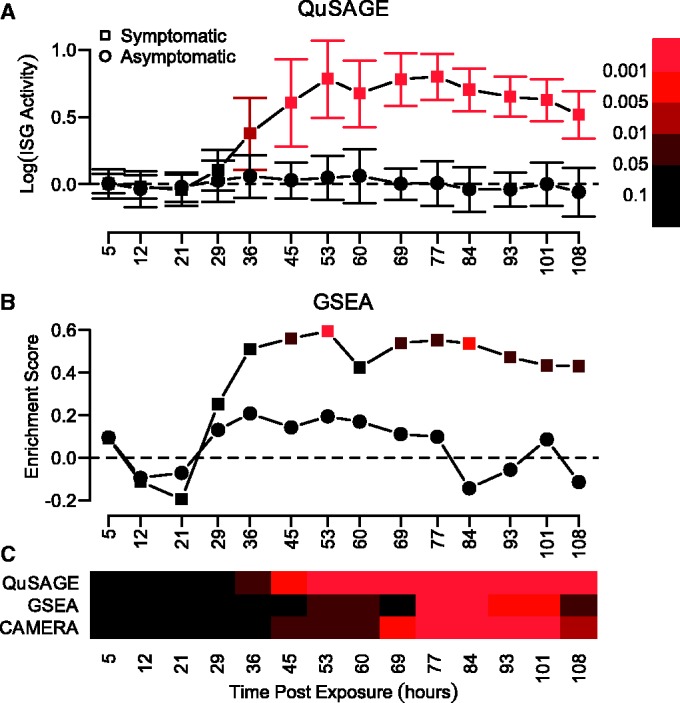
QuSAGE detects earlier and more significant ISG activity in symptomatic (versus asymptomatic) human subjects following influenza exposure. ISG activity was quantified at each time point using (**A**) QuSAGE and (**B**) GSEA. Color-coding indicates the *P*-values for detecting activity in asymptomatic (circles) and symptomatic (squares) subjects relative to pre-exposure levels. (**C**) ISG activity was compared directly between the asymptomatic and symptomatic subject groups using QuSAGE, GSEA and CAMERA. Color-coding indicates the *P*-values using the same color key as panels (A) and (B). QuSAGE and CAMERA both estimate the average activity using the same statistic, although the *P*-values can differ.

QuSAGE was also used to quantify ISG activity in asymptomatic and symptomatic subjects following influenza infection. In a previous study, these genes were specifically associated with symptomatic infections (20). Briefly, 17 healthy human subjects were exposed to live influenza and classified as asymptomatic or symptomatic based on the severity of symptoms. Peripheral blood was collected at approximately 8 h intervals up to 108 h post-exposure for gene expression analysis. To compare the sensitivity of QuSAGE with existing methods (GSEA and CAMERA), we quantified the activity of ISGs at each time-point relative to the pre-exposure levels (Figure 7A). As expected, all approaches generally showed stronger ISG activity in symptomatic patients. However, while the qualitative activity patterns were similar, QuSAGE was able to detect statistically significant activity (*P* < 0.05) at earlier time points (36 h post-exposure for QuSAGE and CAMERA versus 45 h for GSEA). The *P*-values produced by QuSAGE were also consistently smaller. QuSAGE was also able to detect stronger and earlier differences in ISG activity when comparing asymptomatic and symptomatic subjects directly to each other (36 versus 45 and 53 h post-exposure, for QuSAGE, CAMERA and GSEA, respectively) (Figure 7C). Although none of the approaches detected significant activity in asymptomatic subjects, the activity estimated by QuSAGE was much smoother and closer to zero compared with GSEA (compare Figure 7A and B). Thus, QuSAGE exhibits increased sensitivity compared with both GSEA and CAMERA.

## DISCUSSION

QuSAGE is a statistical framework for gene set analysis. Focusing on gene sets, rather than individual genes, has proved to be highly useful in practice, and dozens of methods have been proposed (2). Compared with existing approaches, QuSAGE improves power by more accurately accounting for inter-gene correlations while also providing a more intuitive means to estimate gene set differential expression by considering the full PDF confidence interval estimation. This enables post hoc comparisons between gene sets, along with more intuitive visualizations (Figure 5).

Many existing methods for gene set analysis fail to account for prevalent inter-gene correlations, resulting in a high Type I error. One approach to account for the lack of gene independence is to compute *P*-values by using a permutation of sample labels to generate the null distribution, as employed by the widely used GSEA (15). However, this can be computationally intensive and has also been criticized for mixing null hypotheses (6). In addition, permutations require a large number of samples in each group. When the number of samples per group is less than seven, GSEA suggests switching to gene set permutations, which again has the problem of assuming gene–gene independence. Moreover, if multiple hypothesis corrections are to be applied, the minimum sample number increases. A more recent approach, CAMERA, attempts to correct for inter-gene correlations by estimating a VIF directly from the data (13). QuSAGE builds on this approach but corrects for a number of shortcomings including the assumptions that: (i) the standard deviation for individual genes is the same across groups (i.e. treatment and control) and (ii) all genes have the same variance. This first assumption is also made by many popular methods for differential expression analysis, including LIMMA (23), which make use of the pooled variance in calculating statistics. However, we find little evidence to support this assumption and rather observe that standard deviations for many genes can differ significantly across groups in real data sets. Although QuSAGE can work with the pooled variance, we recommend the use of the Welch approximation, which does not assume equal variance.

Although QuSAGE does not require as many samples as permutation-based methods, the current implementation requires at least three degrees of freedom. When using the pooled variance, it is possible to analyze data with two samples in each group (degrees of freedom is 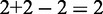). However, as the variance of a t distribution is 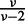, it diverges for 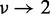. Hence, in cases where the degrees of freedom falls below three, QuSAGE artificially set the degrees of freedom to be three (In this case, the QuSAGE implementation also issues a warning message that the results should be interpreted with caution.). Small numbers of samples can also add significant noise to the estimation of gene–gene correlations, and this impacts the VIF calculation. QuSAGE estimates these correlations directly from the data, but this is not strictly necessary. Assuming that these correlations reflect non-specific co-regulation (13), they could be estimated from other experiments. Indeed, databases of gene–gene correlations have been developed (31), and this information could be directly incorporated into the QuSAGE VIF calculation to improve the analysis for data sets with a small number of samples.

QuSAGE quantifies gene set activity as a shift in the mean differential expression of genes that compose the set. One might think that this approach could weaken the biological signal by averaging strongly differentially expressed genes with weakly differentially expressed genes and thus diminish the estimated statistical significance. However, while including ‘weak’ differentially expressed genes does indeed lower the activity (by definition, as activity is simply the average fold-change of genes in the set), the statistical significant actually increases. To demonstrate this behavior, we used the data in Figure 5A to re-estimate ISG set activity as ‘weak’ genes were added to the gene set. As seen in Supplementary Figure S2A, adding genes that are less differentially expressed does lower the activity (as expected by the definition of activity). However, the overall statistical significance actually increases (see Supplementary Figure S2B). Thus, rather than exhibiting a ‘dilutional’ effect, QuSAGE benefits from the inclusion of all genes. For gene sets that include both up- and downregulated genes, this approach may not be sufficient (32). In cases where the directionality of individual genes is known (e.g. based on prior biological knowledge or because the set was based on observed gene expression changes), then the signs of these expectations can be used to modify the direction of the corresponding individual genes differential expressions in Equation (6). When such knowledge is not available, the QuSAGE package offers a method to combine individual genes *P*-values within a set. First, *P*-values are computed for each gene by comparing the differential expression PDF with either: (i) zero, (ii) the mean activity of the gene set or (iii) the full PDF of the entire set. These *P*-values are then combined into one score for the gene set using the Brown method (33), which accounts for gene–gene correlations by exploiting information from the correlation matrix (derived from the covariance matrix in Equation (11)). We have validated this method using the approaches shown in Figures 3 and 4, and confirmed that the test is conservative (data not shown). However, this method loses the benefit of estimating the full activity PDF. Gene sets that include both up- and down-regulated genes are also a problem for the widely used GSEA method. In this case, it has been proposed to rank genes by the absolute value of their signal-to-noise ratio (or other statistic used for ranking) (15). A similar approach could be adapted here by creating a full confidence interval PDF of the absolute value difference between two groups before the convolution step. However, in this case, the individual gene PDFs are no longer symmetric, requiring an alternate convolution technique and method to account for gene–gene correlations.

Like most gene set analysis methods, QuSAGE assumes that log expression values are normally distributed. This is generally accepted for gene expression microarray data. The normality assumption also holds for RNA-seq data when the number of samples is sufficiently large (34), and QuSAGE can be directly applied to expression values calculated from normalized RNA-seq counts. However, for small numbers of samples, RNA-seq data are better described using a negative binomial distribution, whose parameters can be estimated from the entire data set by several approaches (35–37). QuSAGE could be adapted to this case by replacing the t-distribution with a negative binomial distribution to model individual genes.

An implementation of QuSAGE is made available as an R package, which can be downloaded from http://clip.med.yale.edu/QuSAGE. Significant work has been done to optimize the code for computational efficiency. The current implementation can be used to analyze data in real time on a standard desktop computer. For example, the entire analysis that is shown in Figure 5C took < 20 s on a single 2.70 GHz processor. This involved assessing 186 gene sets (typically 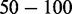 genes) on a data set composed of 10 subjects with 19 798 genes measured at two time points. Thus, QuSAGE provides an efficient means to quantify, analyze and visualize gene set activity.

## SUPPLEMENTARY DATA

Supplementary Data are available at NAR Online.

## FUNDING

Funding for open access charge: NIAID contract [HHSN272201000054C]; NIH (in part) [T15 LM07056 to C.R.B.].

*Conflict of interest statement*. None declared.

## Supplementary Material

Supplementary Data
