# Supplementary material for: Quantitative set analysis for gene expression: a method to quantify gene set differential expression including gene-gene correlations
Source: Nucleic Acids Res. 2013 Aug 5;41(18):e170. doi: 10.1093/nar/gkt660 (PMC3794608; doi:10.1093/nar/gkt660)
Supplement: Supplementary Data [file supp_41_18_e170__index.html]

Quantitative set analysis for gene expression: a method to quantify gene set differential expression including gene-gene correlations — Quantitative set analysis for gene expression: a method to quantify gene set differential expression including gene-gene correlations — Supplementary Data 

# Quantitative set analysis for gene expression: a method to quantify gene set differential expression including gene-gene correlations

## 

files

**Files in this Data Supplement:**

- Supplementary Data - pdf file
