# Supplementary material for: Quantitative set analysis for gene expression: a method to quantify gene set differential expression including gene-gene correlations
Source: Nucleic Acids Res. 2013 Aug 5;41(18):e170. doi: 10.1093/nar/gkt660 (PMC3794608; doi:10.1093/nar/gkt660)
Supplement: Supplementary Data [file supp_gkt660_nar-00767-met-n-2013-File016.pdf]

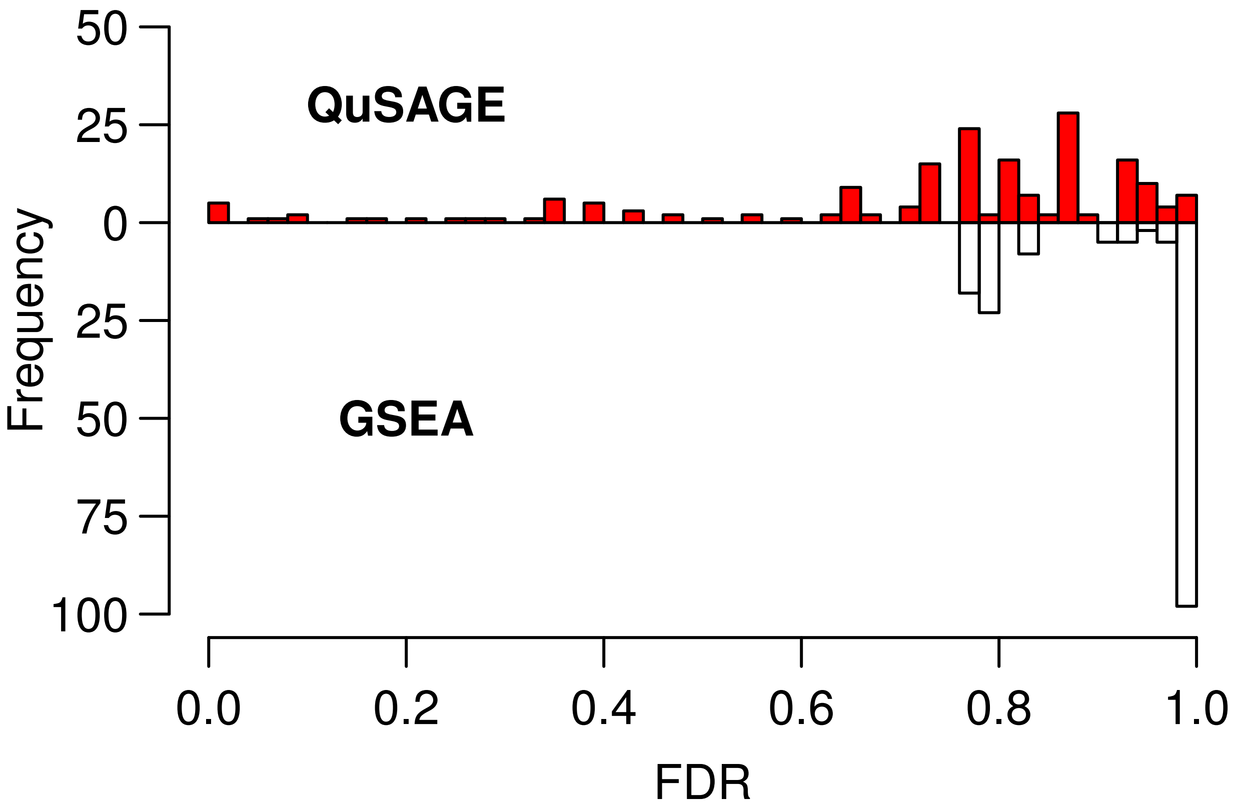

Figure S1: **Improved sensitivity of QuSAGE compared with GSEA on a dataset with 10 samples**. The activity (post- versus pre-therapy) of 186 pathway gene sets from KEGG was analyzed in gene expression data from clinical responders in an HCV patient cohort (same data used in Figure 5B). FDR corrected P values are shown for QuSAGE (red bars) and GSEA (open bars).

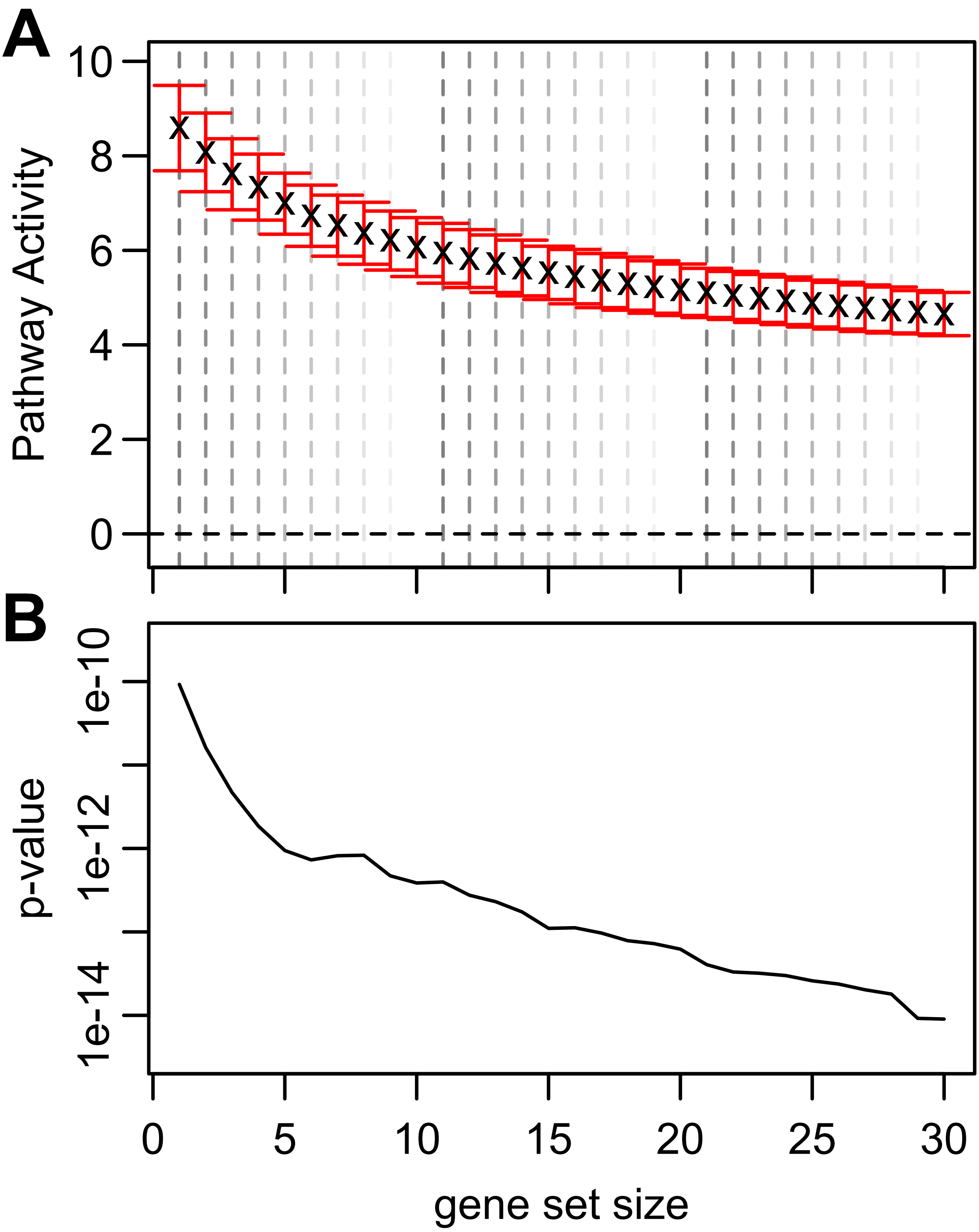

Figure S2: **Effect of adding “weakly” differentially-expressed genes to the gene set**. Genes in the ISG pathway were ranked according to their estimated activity in clinical responders (post- versus pre-therapy) using the data from Figure 5A. Starting with the most active, gene sets were created by successively adding weaker genes (X axis). (A) QuSAGE was used to quantify activity for each set. (B) The resulting P value for detecting significant activity compared to zero (in logarithmic scale).
